# Polyuria

**Published:** 1908-05-02

**Authors:** 


### Diseases of Children.

### POLYURIA.

The frequent passage of urine by nurslings is not in itself a proof of polyuria, though even true diabetes insipidus may start in early life. Normally an infant passes urine from six to ten times in twenty-four hours, usually after being fed or on waking from sleep. At such times, by attention to the baby, it is possible to obtain the urine in a suitable receptacle for examination. Bottle-fed infants may wet themselves every half-hour. If so, it is generally found that the diet consists of condensed milk or a dilute fluid food rich in sugar. Excessive frequency of micturition is sometimes found in the breast-fed when the mother's milk contains an unduly high percentage of milk sugar. Barley water may have a similar effect.

After infancy polyuria is usually due to cold weather, insufficient clothing, excess of fluid ingested, digestive disturbance, emotional disturbance, local irritation, or serious disease, such as diabetes mellitus and chronic interstitial nephritis. It may also result from the absorption of dropsical effusions or the action of diuretics and of drugs, such as digitalis. Again, it is imperative to distinguish polyuria from undue frequency of micturition. The total amount of urine passed and of fluid ingested in the twenty-four hours should be measured.

Polyuria and increased frequency of micturition are almost invariably associated, whereas increased frequency does not necessarily mean polyuria, though there is generally some increase in the total daily excretion. As a rule, the child comes under observation for increased frequency of micturition or for enuresis, and unless a thorough examination is made there is considerable liability to error in treatment. In every case of enuresis it is of importance to exclude the presence of those serious diseases, true diabetes and chronic interstitial nephritis, both of which are distinctly uncommon in early life, and are fairly easy of diagnosis when suspected.

True diabetes insipidus is said to be a rare disease in early life, though it is probably as common in children as in adults, and may even date from infancy. On the whole, it is better to regard the affection as a special entity, dependent on a nervous causation, and to differentiate it thus from the polyuria of some hysterics. There is no doubt that it is a hereditary neurosis, and it may affect many members in the same family. Cases have followed injury—*e.g.*, blow on the left temple; fracture of the base of the skull; and fracture of the skull in association with hemianopsia in a girl aged fourteen years. Others have followed on specific fevers, or have been due to cerebral tumour, while in many no cause can be found.

The amount of urine varies considerably, from 5 to 20 pints, with a specific gravity of 1001-1006, pale colour, and containing neither albumin nor sugar. Inosite has been found in a few cases.

In a typical case we find that the patient is a thin neurotic girl with a dry mouth and unduly red tongue, a dry harsh and pigmented skin, headache, thirst, frequent micturition, enuresis, and symptoms of neurasthenia. Sometimes the appetite is said to be increased. The course is indefinite. Some recover spontaneously. The prognosis depends on the cause, duration, and the presence of organic disease. It is best when the polyuria is a sequel of injury or fever, and worst when there is a strong family predisposition. Temporary improvement and periodical relapses are common. As an instance of the difficulty in diagnosis may be mentioned a case reported in the *Lancet* a few years ago. The child, a girl of eight, died in coma two weeks after the onset of polyuria and polysitism. Urine 1008, quantity 6 to 8 pints, trace of albumin. There was no autopsy. The report suggests that this was really a case of chronic interstitial nephritis.

The pathology of true diabetes insipidus is uncertain. Probably there is a defect in the function of the kidneys, rendering them unable to excrete urine above a certain degree of concentration, which is usually much less than normal. Consequently an excess of fluid must be ingested. If the amount is diminished, the urinary solids are not sufficiently excreted, and symptoms like uræmia arise. Another view is that the mechanism of concentration is at fault, and that probably the defect is in the floor of the fourth ventricle. An increase of the salts in the food leads to increased polyuria, for the specific gravity of the urine remains the same. Apart from theoretical considerations, clinical observations show that much good can be done by treatment in many cases, while in others the affection is more or less constant throughout life. It is of doubtful advantage, and may be distinctly dangerous to restrict fluids severely, for the result may be impaired digestion, refusal of food, irritability, and symptoms suggestive of uræmia. The diet should be simple and nutritious, and the different food stuffs proportionately regulated. Neither excess nor reduction of meat has in my hands proved curative. General hygiene and bathing must be attended to. Of drugs the most benefit will be derived from valerian, antipyrin, bromides, belladonna, and arsenic. Occasionally ergot and nitric acid are useful. Valerian must be given in large doses, especially in purely nervous cases. It can be given in the form of valerianate of zinc, made into a pill with powdered ginger and rhubarb and extract of gentian. Antipyrin comes next in value, also in large doses. It not only reduces the reflex excitability of the cord, but also the excretion of the urine, and is consequently of value in organic nerve lesions and in enuresis. It may be injurious if there is renal disease. Belladonna and atropine are valuable, especially so in enuresis of nervous origin. A good way of prescribing atropine is to give one minim of the liquor at four and seven P.M. and to increase the dose by one minim only per day until the child takes as many minims as it is old in years. Cold douching to the spine while the child stands in a tub of hot water is also useful. Finally, if all else fails, it is worth while trying the effect of faradism of the lumbar region or systematic galvanism of the spine.

